# Nivolumab-induced autoimmune diabetes mellitus presenting as diabetic ketoacidosis in a patient with metastatic lung cancer

**DOI:** 10.1186/s40425-017-0245-2

**Published:** 2017-05-16

**Authors:** James Luke Godwin, Shuchie Jaggi, Imali Sirisena, Pankaj Sharda, Ajay D. Rao, Ranee Mehra, Colleen Veloski

**Affiliations:** 1 Department of Hematology/Oncology, Fox Chase Cancer Center, Philadelphia, PA USA; 2 Department of Medicine, Section of Metabolism, Diabetes and Endocrinology, Temple University Hospital, Philadelphia, PA USA; 3 Department of Medicine, Section of Endocrinology, Fox Chase Cancer Center, Philadelphia, PA USA; 4 Department of Oncology, Johns Hopkins Hospital/Sidney Kimmel Comprehensive Cancer Center, Bloomberg~Kimmel Institute for Cancer Immunotherapy, Baltimore, MD USA

**Keywords:** PD-1 inhibitor, Nivolumab, Non-small cell lung cancer (NSCLC), Immune related adverse events (irAE), Autoimmune diabetes, Diabetic ketoacidosis (DKA)

## Abstract

**Background:**

Advances in cancer immunotherapy have generated encouraging results in multiple malignancies refractory to standard chemotherapies. As the use of immune checkpoint inhibitors (ICI) proliferates, the incidence of autoimmune side effects associated with these agents, termed immune related adverse events (irAE), is expected to increase. The frequency of significant irAE in ICI treated patients is about 10–20% and early recognition is critical to prevent serious morbidity and even mortality. New onset autoimmune diabetes mellitus (DM) associated with immune checkpoint inhibitor treatment is extremely rare, occurring in less than 1% of patients. Autoimmune DM often presents as diabetic ketoacidosis, a medical emergency requiring immediate treatment. We describe the first reported case of a patient with lung cancer who developed autoimmune diabetes after nivolumab treatment and was found to have three diabetes related (islet) autoantibodies present before ICI treatment and seroconversion of another after ICI treatment and onset of autoimmune DM.

**Case Presentation:**

A 34 year old African American woman with metastatic non-small cell lung cancer (NSCLC) was treated with nivolumab in the second line setting after disease progression following standard chemoradiation therapy. After receiving two doses of nivolumab, the patient developed abrupt onset of hyperglycemia and diabetic ketoacidosis. Autoimmune diabetes was diagnosed on the basis of undetectable C-peptide levels, seropositivity of three diabetes related (islet) autoantibodies and absolute insulin dependence. The patient eventually required use of continuous subcutaneous insulin infusion (insulin pump) due to erratic glycemic excursions and multiple readmissions for DKA. Human leucocyte antigen (HLA) genoyping revealed none of the high risk haplotypes associated with the development of type 1 diabetes. Interestingly, a frozen blood sample obtained prior to treatment with nivolumab tested positive for three of the four diabetes related (islet) autoantibodies despite no prior history of diabetes and no family history of diabetes. Notably, at the time of manuscript preparation, the patient is without evidence of NSCLC recurrence with no further treatment since the nivolumab therapy.

**Conclusion:**

New onset autoimmune diabetes mellitus associated with nivolumab has been described only in case reports and occurs at rates of < 1% in the large clinical trials which garnered FDA approval in the second line setting for NSCLC. As ICI use continues to expand across a wide variety of malignancies, clinicians must maintain a high index of suspicion for irAE, including autoimmune DM and other endocrinopathies. A multidisciplinary team and thorough education of the patient are recommended to optimize management of new onset adult autoimmune DM. Our patient may have been at greater risk for the development of ICI related autoimmune diabetes due to the presence of three diabetes related autoantibodies prior to therapy; however, about half of the reported cases of autoimmune DM after anti-PD-1 therapy occurred in patients with no detectable diabetes related autoantibodies. Further studies are needed to delineate genetic and immunologic biomarkers that may be useful in identifying patients at risk of developing ICI related autoimmune DM.

## Background

Immunotherapy represents one of the most exciting areas of therapeutic advances and research in oncology today. Immune checkpoint inhibitors (ICI) are drugs which disrupt inhibitory signaling to T cells, thus potentially activating and augmenting an anti-tumor response. One of the best known checkpoints is “Programmed Death 1” (PD-1), a cell surface protein found on activated T cells which, when bound to its ligands (PD-L1 and PD-L2), inhibits kinase signaling pathways that normally lead to T-cell activation. Within the past 3 years, four monoclonal antibodies targeting the PD-1-PD-L1 axis have been approved by the FDA for use: nivolumab (anti-PD1, approved in melanoma, NSCLC, renal cell carcinoma, Hodgkin lymphoma, head and neck squamous cell carcinoma (HNSCC), urothelial carcinoma), pembrolizumab (anti-PD-1, approved in melanoma, NSCLC, HNSCC, Hodgkin lymphoma,) atezolizumab (anti-PD-L1, approved in urothelial cell carcinoma and NSCLC) and avelumab (anti-PD-L1, approved in Merkel cell carcinoma). Many other agents targeting the PD-1/PD-L1 axis, as well as other immune checkpoints, are currently being studied in phase III trials and future approvals across the spectrum of tumor types are expected within the next few years. As this field continues to expand, clinicians will be charged with managing the immune related adverse events (irAE) associated with ICI. Although relatively few patients (10–20%) develop significant irAE associated with ICI monotherapy, these events (e.g. pneumonitis, colitis) can be serious and life-threatening. Combination ipilimumab/nivolumab therapy has the highest rate of significant irAE (nearly 40%) while the newer anti-PD-1 antibodies such as nivolumab and pembrolizumab have fewer significant irAE (<10%) than either ipilimumab monotherapy or combination therapy [1].

Herein, we report a case of rapid onset autoimmune diabetes mellitus (DM) presenting with diabetic ketoacidosis (DKA) and four positive diabetes related autoantibodies in a woman with NSCLC receiving Nivolumab. Three of the four autoantibodies were present before treatment with Nivolumab and before the onset of autoimmune diabetes.

## Case Presentation

A 34 year-old woman with no history of diabetes presented to her local ER in March 2015 with chest pain. Family history was negative for diabetes. A chest X-ray revealed a left lung mass confirmed on subsequent chest CT to be a left upper lobe mass measuring 5.2 × 3.7 × 3.8 cm with left hilar and AP window lymphadenopathy. A bone scan on 3/19/15 was negative for osseous metastases. She underwent bronchoscopy with sampling of the level 4 L, 7 and 11 L lymph node stations. Samples from stations 7 and 4 L were negative for malignancy, but the 11 L station contained tumor cells consistent with high grade adenocarcinoma. A full body staging PET-CT revealed significant FDG avidity in the primary tumor and the left hilar and AP window nodes, without evidence of distant metastatic disease. A brain MRI on 3/31/15 did not reveal metastatic disease. She underwent a staging mediastinoscopy and bronchoscopy, with a total of 19 nodes sampled from 4R, 4 L and 7 lymph node stations, all negative for malignancy. The patient subsequently underwent treatment with concurrent chemotherapy and radiation therapy for unresectable stage IIIA NSCLC (carboplatin and pemetrexed – 4 cycles, given every 3 weeks from April 2015–June 2015). Follow-up CT imaging on 11/3/15 revealed treatment response in the thorax and a new soft tissue lesion near the right acetabulum concerning for a metastatic implant (1.8 × 2.3 cm). A CT guided biopsy of the right gluteal mass revealed metastatic adenocarcinoma. Molecular testing of the metastatic gluteal lesion identified a TP53 mutation, however, activating EGFR, ALK and ROS1 mutations were not present. A restaging PET-CT scan and brain MRI obtained December 2015 showed the metastasis to the right gluteal region as the only active disease site. The patient subsequently opted for systemic therapy with nivolumab. She received her first treatment with nivolumab 170 mg (3 mg/kg) on 12/14/15 and a second dose on 12/28/15, with no acute complications.

Two weeks after the second nivolumab treatment, the patient presented to a local ER with abdominal pain, nausea and weakness progressively worsening over 3 days. Laboratory evaluation revealed diabetic ketoacidosis (DKA), with a plasma glucose 739 mg/dL, venous pH of 7.12, CO2 11, AG 30, and urine ketones > 80 mg/dL. She was admitted to the ICU for IV fluids, continuous insulin infusion and frequent lab monitoring. A hemoglobin A1c level on 1/12/16 was 7.1% (normal range 4.6–6.1%). C-peptide levels on 1/16/16 (while BG 377 mg/dL) and 1/18/16 (while BG 423 mg/dL), were < 0.1 ng/mL (normal range 0.8–3.85). Further evaluation to establish the diagnosis of autoimmune diabetes (Type 1), provided the following results: glutamic acid decarboxylase 65 (GAD-65) antibody > 30 U/ml (normal < 1.0); tyrosine phosphatase islet 2 antibody (IA-2) 6.1 U/ml (normal < 0.8); insulin autoantibody (IAA) 0.4 U/ml (normal < 0.4). HLA genotyping was homozygous for A30 and DR9. (Table 1) Other endocrine testing showed: normal hypothalamic-pituitary-adrenal function with morning ACTH 24 pg/mL (normal 6–50) and cortisol 10 ug/dL (normal 6.7–22.6); subclinical hyperthyroidism with slightly suppressed TSH 0.21 uIU/mL (normal 0.34–5.6), FT4 1.41 ng/dL (normal 0.58–1.64). Thyroid stimulating immunoglobulins and thyroid autoantibodies obtained after hospitalization were negative.

**Table 1:** Clinical history and key laboratory findings

Age/sex	Primary Diagnosis	Medical History	History Family	Anti-PD1 Drug	Other chemo-toxins	Diabetes presentation	Random C-peptide and BG	Time After PD-1	Ab titers before nivolumab^*^	Ab titers after nivolumab	HLA
34/F	NSCLC	None	No history of DM	Nivolumab	Carboplatin, pemetrexed	DKA, BG 739, HbA1C 7.1%, urine ketones >80 mg/dL	<0.1 ng/mL while BG 377 mg/dL	2 wks	+ GAD65 (> 250), + IA-2 (6.2), - IAA (< 0.4), + ZnT8 (64)	+ GAD65 (> 30)^a^, + IA-2 (6.1), + IAA (0.4), - ZnT8 (13)^b^	A30:01,30:02 (A30) D09:CTZ,09:CTZ (DR9)

Diabetic autoantibodies to GAD65, IA-2, and Insulin Ab were performed at Quest Diagnostics, San Juan Capistrano. Normal GAD65 titers < 0.5 IU/mL, IA-2 Ab < 0.8 U/mL, IAA < 0.4U/mL, and ZnT8 Ab <15 U/mL

*Diabetic autoantibody testing before treatment was performed using a stored frozen specimen obtained at time of lung cancer diagnosis, 8 months prior to Nivolumab treatment

^a^Quest changed GAD65 assay type from RIA to ELISA between the time the before and after treatment specimens were processed

^b^ZnT8 Ab obtained 13 months after the onset of diabetes

Due to the rare development of autoimmune diabetes in this patient, we obtained consent to retrieve and test a small amount of serum previously collected prior to therapy with nivolumab and the onset of autoimmune diabetes. The frozen specimen was tested for diabetes related autoantibodies with the following results: GAD65 Ab > 250 IU/ml (normal < 5.0); IA- 2 Ab 6.2 U/ml (normal < 0.8); IAA < 0.4 U/ml (normal < 0.4); zinc transporter isoform 8 (ZnT8) antibody 64 U/mL (normal <15). Interestingly, three of the four diabetes related autoantibodies were positive 8 months prior to the initiation of treatment with nivolumab and before the onset of diabetes. ZnT8 Ab measured 13 months after diagnosis of diabetes had decreased below the cutoff for positivity (<15) (Table 1).

Glycemic control proved challenging with severe instability of glucose and frequent and unpredictable hypoglycemic and/or ketoacidosis episodes. Over the course of 11 days, the patient’s glucose was gradually controlled with a basal-bolus insulin regimen recommended by endocrinology, and the patient was discharged. Unfortunately the patient had multiple re-admissions due to hyperglycemia and DKA over the ensuing month, ultimately requiring placement of an insulin pump. Despite intensive visits with her endocrinologist and diabetes educators, the patient found the insulin pump too difficult to use, eventually discontinuing it and resuming multiple daily injections. While she continues to have erratic blood sugars with frequent hypoglycemia and hyperglycemia, she has become more adept at managing her diabetes and has not been hospitalized for her diabetes. The current plan is to try to obtain a continuous glucose monitor to help manage the extreme glucose variability.

At the time of follow up with medical oncology in March 2016, the patient was clinically improved with near resolution of pain in the right gluteal region. Re-staging CT Chest/Abdomen/Pelvis with contrast in April 2016 revealed a significant treatment response, with decrease in the size of the primary left upper lobe mass and resolution of the gluteal mass. Given these results, her treatment holiday was continued. Re-staging CT scans in October 2016 revealed no definite evidence of disease. At the time of initial manuscript preparation, she is feeling well, on no antineoplastic therapy.

## Discussion and conclusions

Over the past two years, two anti-PD-1 antibodies [nivolumab (Opdivo, Bristol-Myers Squibb), pembrolizumab (Keytruda, Merck)] and one anti-PD-L1 antibody atezolizumab (Tecentriq, Genentech)] have been approved to treat patients with advanced non-small cell lung cancer (NSCLC). These drugs target receptors which provide inhibitory signals to T-cells, thus amplifying T-cell activity in an attempt to generate anti-tumor immune response. As expected, the side effects associated with ICI are often autoimmune, including pneumonitis, colitis and endocrinopathies (e.g. thyroiditis, hypophysitis). Autoimmune or type 1 diabetes mellitus (T1DM) has rarely been reported as a side effect of anti-PD1 therapy, primarily in case reports [2–10]. T1DM is caused by destruction of pancreatic beta cells by autoreactive T-cells. Non-obese diabetic (NOD) mice have been studied extensively as an experimental model since they feature many aspects of T1DM similar to humans. PD-1 interaction with its ligands PD-L1 and PD-L2 is crucial for regulation of CD4/CD8 auto-reactive T cells. In transgenic mice, PD-1 expression was associated with resistance to the precipitation of autoimmune diabetes; however, blockade of the PD-1/PD-L1 axis caused diabetes in the already pre-diabetic NOD mice [11–13].

Nivolumab was approved by the FDA in 2015 for the second line treatment of advanced NSCLC based on the results of two large randomized open-label phase III trials which demonstrated a survival benefit for patients treated with nivolumab versus docetaxel [14, 15]. The first of these to be published (Checkmate 017) included 135 patients with squamous cell lung carcinoma randomized to treatment with nivolumab or docetaxel. Treatment related adverse events included pneumonitis (5%) and hypothyroidism (4%) [15]. A subsequently published trial (Checkmate 057) included 292 patients with advanced NSCLC randomized to treatment with nivolumab or docetaxel. Treatment related adverse events in this trial included hypothyroidism (7%) and pneumonitis (1%) [14]. No episodes of treated-related hyperglycemia or DKA were noted in either of these studies.

Our patient’s autoimmune diabetes was abrupt in onset and very difficult to control due to the complete absence of insulin secretion as evidenced by undetectable C-peptide levels at the time of diagnosis. Blood glucose levels were normal prior to the initiation the treatment with nivolumab. Two weeks after the first dose of nivolumab, a random glucose was elevated at 193 mg/dL; two weeks after the second dose, the patient presented with DKA, a blood sugar of 739 mg/dL. There was no ‘honeymoon period” of preserved beta cell function and good glycemic control often observed at the onset of juvenile Type 1 DM. At the time of diagnosis of autoimmune diabetes, the HbA1C value was 7.1% correlating with a three month blood sugar average of 154 mg/dL, further suggesting a rapid onset of acute hyperglycemia after treatment with nivolumab. The presence of GAD 65, IA-2, and ZnT8 antibodies prior to treatment may have predisposed the patient to the development of autoimmune diabetes. After treatment nivolumab and at onset of diabetes (after treatment with exogenous insulin), IAA seroconverted from negative to positive, perhaps demonstrating the enhanced immune activation against islet antigens. ZnT8 Ab measured 13 months after onset of diabetes fell below the threshold of positivity in agreement with the findings of Vaziri-Sani et al of declining ZnT8 Ab titers rapidly after onset among a longitudinal cohort of young type 1 diabetes patients [16].

Some HLA-I and HLA-II haplotypes are associated with increased susceptibility to T1DM.

Class II haplotypes HLA-DR3-DQ2 (DR3) and HLA-DR4-DQ8 (DR4) are associated with increased risk of T1DM especially in Caucasians [17]. HLA typing of our patient revealed HLA-I A30 and HLA-II DR9 (Table 1); neither is a high risk haplotype associated with the development of T1DM. In contrast to our findings, several case reports have shown an established high risk allele for T1DM (HLA-II DR4 haplotype) present in the majority of patients for whom HLA typing was available [5, 7, 8, 10] while other case reports did not observe an association between HLA and the development of ICI associated T1DM [4, 6, 9].

In contrast to other autoimmune sequelae of anti-PD1 therapy such as pneumonitis or colitis that are routinely treated with high dose steroids per clinical trial protocol specifications and published guidelines, there is little data regarding the use of steroids in the setting of autoimmune diabetes. High dose steroids are usually avoided since they exacerbate hyperglycemia and complicate the management of insulin dependent diabetes. Standard irAE immunosuppression with prednisolone in an attempt to reverse pembrolizumab-induced T1DM did not salvage beta cell function in one case reported by Aleksova et al. [2]. Current management includes traditional treatment strategies for DKA, including intravenous (IV) insulin therapy, IV hydration and frequent monitoring of labs to correct the anion gap and electrolyte derangements. Co-management and communication between medical oncology and endocrinology is essential to coordinate education and close outpatient follow-up to optimize patient outcomes. Glycemic targets must be individualized to the patient, taking into consideration numerous other factors such as patient’s nutritional status, health literacy and overall prognosis. Development of DKA is not an absolute contraindication to continuing nivolumab therapy in patients with advanced NSCLC once the DM is well-controlled, given the paucity of treatment options for these patients. In keeping with a shared decision making model, careful discussion of risks and benefits between the patient and clinician is required. Of note, our patient has experienced a significant ongoing response to nivolumab and has not received antineoplastic treatment since December 2015.

Since it is not standard practice to check for the presence of diabetes autoantibodies prior to ICI treatment, the pre-treatment diabetes antibody status was typically unavailable for the previously reported cases. However, Lowe et al reported a case of autoimmune diabetes following combination therapy with ipilimumab and nivolumab that demonstrated anti-GAD antibody seroconversion from negative to positive [6]. It is not known whether diabetes antibodies were present prior to ICI treatment in the other reported cases to date. To our knowledge, this is the first reported case to describe a patient who was found to have GAD 65, IA-2 and ZnT8 antibodies prior to treatment with nivolumab who then subsequently developed new insulin antibodies and autoimmune diabetes following treatment. It is likely that our patient was in the “preclinical” phase of the development of autoimmune diabetes characterized by the presence of one or more diabetes related autoantibodies and sufficient beta cell function to maintain euglycemia. PD-1 inhibition may have simply accelerated a pre-existing autoimmune process that ultimately led to the development of T1DM in this patient. Indeed, the presence of both GAD and IA-2 antibodies in first degree relatives of patients with T1DM has been shown to confer a 61% risk of developing TD1M in 10 years [18]. Although our patient did not have any first degree relatives with diabetes, she almost certainly was at increased risk of developing T1DM prior to treatment with nivolumab due to the presence of these antibodies.

Reported cases of T1DM related to anti PD-1 have shown conflicting results regarding the presence of diabetes related autoantibodies after development of T1DM. Hughes et al. reported five cases of new-onset insulin-dependent diabetes after receiving anti-PD1 therapy. Three of the five patients described developed at least one positive diabetes related autoantibody after onset of T1DM [5]. At least half of the reported cases of ICI related autoimmune diabetes show no detectable diabetes autoantibodies at onset [2, 4, 5, 8–10]. Similar results have been found in the NOD mouse model of autoimmune diabetes. Ansari and colleagues found no correlation between insulin autoantibody levels and development of autoimmune diabetes in NOD mice treated with PD-1–PD-L1 blockade; some mice developed diabetes with no antibodies and others developed antibodies but did not develop diabetes [13]. Similarly, HLA alleles associated with increased risk of T1DM have been present in some but not all reported cases of ICI related T1DM.

As the approved indications for anti-PD1 therapy expand in the coming years, the number of patients receiving these drugs will dramatically increase, as will the frequency of autoimmune adverse events. Medical oncologists must be aware of the possibility of anti-PD1 therapy induced autoimmune diabetes and counsel their patients to report symptoms that may be related to DKA, as this is a medical emergency. The presence of diabetes autoantibodies prior to anti-PD1 therapy likely predisposed our patient to developing T1DM. It is possible that the presence of diabetes antibodies is a risk factor for ICI related T1DM. However, the absence of diabetes autoantibodies after onset of T1DM in some cases of ICI related T1DM suggests other factors are involved. Future correlative studies in this small patient population are needed to delineate genetic and immunologic biomarkers to identify those at highest risk for autoimmune sequelae, such as autoimmune DM.

## Abbreviations

Ab: Antibody
DKA: Diabetic ketoacidosis
DM: Diabetes mellitus
FDA: Food and Drug Administration
GAD: Glutamic acid decarboxylase
IA2: Tyrosine phosphatase islet antigen 2
IAA: Insulin autoantibody
ICI: Immune checkpoint inhibitors
irAE: Immune-related adverse events
NOD: Non-obese diabetic
NSCLC: Non-small cell lung cancer
PD-1: Programmed Death 1
PD-L1: Programmed Death Ligand 1
ZnT8: Zinc transporter isoform 8
